# Micromachines Beyond Silicon-Based Technologies: A Letter from the New Editor-in-Chief

**DOI:** 10.3390/mi7030044

**Published:** 2016-03-09

**Authors:** Nam-Trung Nguyen

**Affiliations:** Editor-in-Chief of *Micromachines*, Queensland Micro- and Nanotechnology Centre, Griffith University, 170 Kessels Road, Brisbane, Queensland 4111, Australia; nam-trung.nguyen@griffith.edu.au; Tel.: +61-7-3735-3921; Fax: +61-7-3735-8021

It is my pleasure to assume the role of the Editor-in-Chief of *Micromachines* from March 2016. The journal was founded in 2009 by Prof. Miko Elwenspoek from the University of Twente, and published its first issue in 2010. Twenty years ago, I spent 6 months as a visiting PhD student in Prof. Elwenspoek’s research group of Transducers Science and Technology, working on thermal flow sensors. Taking over the responsibility from Prof. Elwenspoek is my personal honour that represents a true generation change in the leadership of our editorial board.

Under Prof. Elwenspoek’s leadership, the journal has grown in both quantity and quality over the past five years. The number of submissions has almost doubled on a year-to-year basis. In 2015, even with a rejection rate of 52%, the number of published papers increased by 50.5% as compared to 2014. The journal has been indexed in all major databases and reached an impact factor of 1.269 (2014). As of March 2016, the journal has published 386 papers with 895 citations (778 citations without self-citations) and an H-index of 13. This performance is truly remarkable for our relatively young journal. The accomplishment was not possible without the leadership of the former Editor-in-Chief and the dedication of the highly efficient team in the editorial office. I would like to take this opportunity to thank our editorial office team for their wonderful support and dedication.

The aim of *Micromachines* is serving the research community with an international, peer-reviewed and open-access forum for studies on micro- and nanoscale machines. Thus, traditionally the journal covers a range of topics related to Micro Electro Mechanical Systems (MEMS), Nano Electro Mechanical Systems (NEMS), Lab on a Chip (LOC), and other microdevices as well as their applications. Most of these systems and devices come from the legacy of silicon-based microtechnologies established over the past fifty years. To further improve the impact of the journal and to reach a broader audience, my vision for the journal is to broaden the subject areas beyond silicon-based microsystems and microdevices. Examples for this broader approach are bio-related applications, bio-inspired design of micromachines, micro and nanomachining of non-silicon materials, bottom–up construction of micro- and nanomachines. In fact, papers published in the past year already evidently supported this trend.

An excellent example for biomedical applications of micromachines is the Special Issue “Biomedical Microdevices” that consists of 14 high-quality papers [1]. The issue reports the design, fabrication and test of typical microfluidic devices for sample preparation such as micromixers [2], micropumps [3,4], and micro concentration gradient generators [5]. The matching size scale makes micromachines attractive tools for cell handling such as cell stretchers [6], and cell separators [7].

The design of micromachines can find inspiration from nature, as natural systems are optimised machines perfected by millions of years of evolution. The bionic mimosa robot [8] and the insect-inspired micropump [9] reported last year was good examples for bio-inspired designs. In terms of micromachining technology for non-silicon materials, machining of aluminium nitride, a seminconducting material with wide band gap [10,11], opens up interesting applications for high-power, high-temperature MEMS devices. Soft microsystem is another emerging topic, where device materials are often made of polymers. Polymeric micromachining is therefore an interesting topic of the future [12,13].

An enthusiastic editorial board of renowned researchers representing the broad topics and interests of the journal currently supports *Micromachines*. I’m honoured to work with this outstanding editorial team as well as the dedicated team of the editorial office. Our collaborative effort will surely bring the journal to the next level of quality and quantity in the years to come. The year 2016 will not disappoint readers of *Micromachines*. We have a line-up of various exciting Special Issues reporting emerging topics such as three-dimensional (3D) printing, nanomaterials for MEMS, bio-inspired systems, bottom–up self-assembly, polymeric technology and microfluidic bioreactors. I am looking forward to the upcoming exciting journey with contributors and readers of *Micromachines*.
